# The “Super Chimpanzee”: The Ecological Dimensions of Rehabilitation of Orphan Chimpanzees in Guinea, West Africa

**DOI:** 10.3390/ani3010109

**Published:** 2013-02-06

**Authors:** Lissa Ongman, Christelle Colin, Estelle Raballand, Tatyana Humle

**Affiliations:** 1 DICE, School of Anthropology and Conservation, Marlowe Building, University of Kent, Canterbury CT2 7NR, UK; E-Mail: lissaongman@googlemail.com; 2 Project Primates France, 140 Residence Boisserette, Rue du Stade 73190, France; E-Mails: wara_guinee@yahoo.fr (C.C.); esthel@yahoo.com (E.R.); 3 Chimpanzee Conservation Center, Faranah, B.P. 36, Republic of Guinea

**Keywords:** chimpanzee, rehabilitation, socially-biased learning, abnormal behaviors, reintroduction, sanctuary, bush-outings

## Abstract

**Simple Summary:**

This study examines relevant behavioral indicators of rehabilitation success of orphaned chimpanzees, victims of the bushmeat and pet trade, and contributes to identifying future release candidates. Results highlight the importance of bush-outings in the development of species-specific behaviors. Neither trauma upon arrival nor contact with human caretakers predicted dietary knowledge among rehabilitants at the Chimpanzee Conservation Centre where the study took place. The studied orphans demonstrated a relatively broad dietary knowledge. We attributed this result to the combined effect of the multiregional origins of residents and the learning opportunities available during bush-outings, which we termed the “Super Chimpanzee” theory.

**Abstract:**

To date few studies, especially among non-human primates, have evaluated or monitored rehabilitation effectiveness and identified key species-specific behavioral indicators for release success. This four-months study aimed to identify behavioral indicators of rehabilitation success among ten infant and juvenile orphaned chimpanzees cared for in peer groups at the Centre for Conservation of Chimpanzees (CCC), Guinea, West Africa. Behavioral data focused on foraging skills and activity budget. During bush-outings, rehabilitants spent on average nearly a quarter of their activity budget foraging, resting or traveling, respectively. Neither age, sex, the level of abnormal behaviors demonstrated upon arrival nor human contact during bush-outings predicted individual dietary knowledge. However, individuals who spent more time arboreal demonstrated a greater dietary breadth than conspecifics who dwelled more terrestrially. Although our data failed to demonstrate a role of conspecific observation in dietary acquisition, we propose that the mingling of individuals from different geographical origins may act as a catalyst for acquiring new dietary knowledge, promoted by ecological opportunities offered during bush-outings. This “Super Chimpanzee” theory opens up new questions about cultural transmission and socially-biased learning among our closest living relatives and provides a novel outlook on rehabilitation in chimpanzees.

## 1. Introduction

The persistence of habitat destruction and the bushmeat and pet-trade across wild chimpanzee range states has led to a marked increase in centers or sanctuaries caring for orphaned chimpanzees [1,2,3,4,5,6,7]. The Pan African Sanctuary Alliance (PASA) was created in 2000 in response to this conservation and welfare urgency, all the more exemplified by a 15% annual growth rate of sanctuary residents between 2000 and 2006 across PASA-member sanctuaries [6,8]. Due to what some refer to as a ‘moral’ and “ethical” obligation to the welfare of individuals who have been taken from their natural social and environmental settings, this situation has led to the creation of multiple chimpanzee sanctuaries across Africa [6]. Sanctuaries provide conditions for rehabilitation, which contribute to improving both an individual’s welfare and wellbeing by promoting species-specific behaviors. In this context, rehabilitation can be defined as “the process by which captive (primates) are treated for medical and physical disabilities until they regain health, are helped to acquire natural social and ecological skills, and are weaned from human contact and dependence, such that they are able to survive in the wild” ([9], p.5). This multifaceted and sometimes complex and lengthy process is rendered even more challenging when it entails additionally overcoming sometimes serious individual trauma frequently associated with capture and mistreatment at the hands of previous owners [10]. 

The developmental trajectory of young chimpanzees and other non-human primates is often affected by exposure and response to learning opportunities, which may be constrained by the individual’s ecological and social environment and psychological state [11,12,13]. Solitary confinement and early maternal removal can lead to more timid personalities, less social activity, less dominance and more susceptibility to stress in orphan chimpanzees [14]. One approach to decreasing the expression of aberrant or stereotypic behaviors is through environmental enrichment [15]. Many PASA-member sanctuaries often rehabilitate individual orphans in large naturalistic enclosures that encourage a natural fission-fusion group lifestyle either in peer-aged or multi age-class social groupings with multi-males and multi-female composition [16]. In these highly enriched environments, Wobber and Hare [16] showed that sanctuary bonobos *(Pan paniscus*) exhibited higher levels of cognition when tested on an assortment of cognitive tasks and a lower level of abnormal behaviors than their captive zoo counterparts. 

Some sanctuaries are able to carry out bush-outings as part of their rehabilitation program; these usually benefit all or a subset of their younger residents. Bush-outings aim to enhance and develop species-specific behaviors such as nesting skills, anti-predatory behaviors, social interactions, food selection and processing. For instance, the diet provided in most captive environments does not encompass the wide range of floral and faunal species consumed typically in the wild, and individuals potentially destined for release need to recognize and process natural foods to survive independently of human provisioning [3]. Bush-outings also expose individuals to natural three-dimensional spaces creating opportunities for locomotion and for dietary knowledge expansion. In the wild, chimpanzees spend a large proportion of daylight hours foraging, *i.e.*, selecting and manufacturing tools to access embedded foods, and/or selecting and processing food items for consumption [17,18]. Wild chimpanzees consume mostly fruit, although their diverse food repertoire also often includes leaves, flowers, bark, stems, seeds, honey, insects and small- to medium-sized mammals [19,20,21,22]. It is from infancy through adolescence that wild chimpanzees have the opportunity to acquire social and foraging skills necessary for adult life [23,24,25,26]. It is important that these social and foraging skillsets develop evenly allowing no deficit in either vital domain of behavior in adulthood. 

In order to achieve a comprehensive foraging repertoire, immature primates can benefit from observing their mother, as well as older and more experienced members of their community and from socially-biased and environmental learning opportunities [27,28,29,30,31]. It is therefore vital during rehabilitation to promote an environment conducive to socially-biased learning and offer multiple learning opportunities for rehabilitant individuals to acquire the skills necessary for survival in the wild. In sanctuaries, where infant and juvenile chimpanzees are without a mother, orphans can learn socially essential survival skills by paying attention to typically unrelated conspecifics and in some cases their human caretakers. Since weaning in the wild promotes independent social and ecological behaviors [24], it is also critical to mimic weaning from human contact in sanctuary settings. Dependency on human contact after a certain age could potentially compromise the emotional, nutritional and social independence of an individual and hinder his/her appropriate social integration with conspecifics [32]. Dependency on human contact could also impact release success if released individuals seek human presence at the expense of exhibiting naturalistic species-specific social behaviors and interactions with conspecifics [9].

Research into assessing effective rehabilitation processes for sanctuary chimpanzees and determining criteria of rehabilitation and release success is currently lacking. Since rehabilitants were not constrained by food availability since provisioned when enclosed, this study aimed to first test whether there was a trade-off between social and foraging domains of behavior during bush-outings. We then analyzed the activity budgets of rehabilitated chimpanzees on bush-outings and assessed individual’s dietary knowledge. This study also sought to examine the influence of abnormal behaviors upon arrival, sex and age and other relevant variables on individual’s dietary acquisition (*i.e.*, observation of conspecifics foraging) and knowledge. Finally we also investigated whether human contact negatively impacted dietary knowledge and observation of the foraging behavior of conspecifics. 

## 2. Methods

### 2.1. Study Site, Subjects

**Figure 1:**
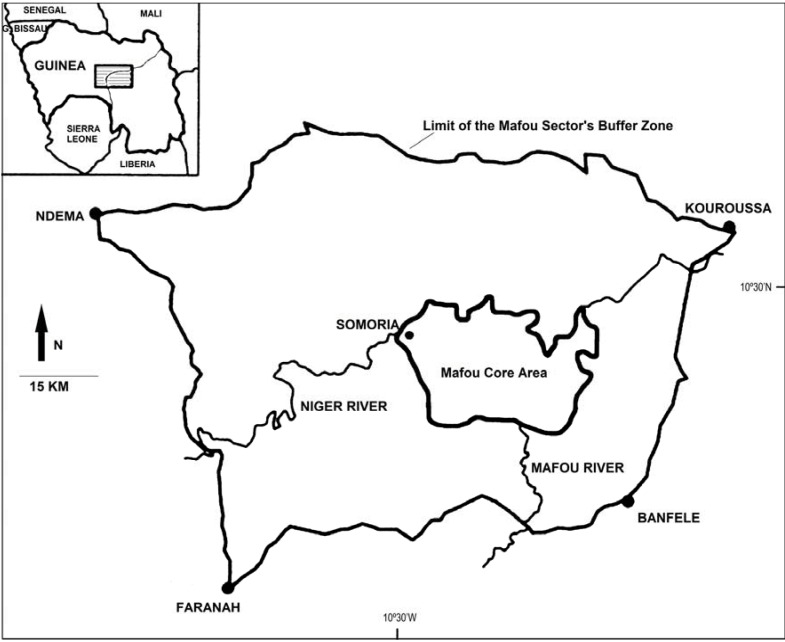
Map of the location of the Haut Niger National Park (HNNP) in Guinea, West Africa, highlighting the Mafou core area of the park and the relative location of the sanctuary in Somoria (adapted from [33]).

All data were collected at the Centre for Conservation of Chimpanzees (CCC) in Somoria, Guinea, West Africa (Figure 1). The CCC is the sole PASA-member sanctuary recovering orphan chimpanzee (*Pan troglodytes verus*) victims of the bushmeat and pet trade in Guinea. This sanctuary currently cares for 41 chimpanzees. Since the national legislation states that it is illegal to kill or possess a chimpanzee, individual chimpanzees, most often infant victims of the pet trade, are typically confiscated by relevant government authorities and handed over to the CCC, where their care and wellbeing in a semi-natural environment is ensured. Individuals come from all over Guinea and occasionally neighboring countries. The CCC is located at the northeastern edge of the Mafou core area of the Haut-Niger National Park (HNNP), one of the last remaining dry forest-savanna mosaics in West Africa [33]. The HNNP harbors open savanna, wooded savanna, bamboo forest, open dry forest, and riparian forest [34].

After a three months quarantine, new arrivals are introduced to peer groups. Study individuals were housed and rehabilitated in two separate peer groups—the “TDT” and the “Nursery”. At the time of the study, the “TDT” group contained three chimpanzees (one male, two females, average age = 3.67 yrs ± 1.53) whereas the “Nursery” group comprised seven chimpanzees (four males, three females, average age = 6.9 yrs ± 0.89) (Table 1). The average age upon arrival for all subjects was 1.8 yrs ± 0.92 (Table 1). Each individual had been rehabilitated for at least a year, and many had been cared for at the CCC for the majority of their infant lives (average time at sanctuary: “TDT” = 1.67 yrs ± 1.15, “Nursery” = 5.15 yrs ± 1.07) (Table 1). Upon confiscation, the geographical origin of capture was confirmed for five of the individuals (Fouta Djallon (n = 2), High Guinea (n = 1) and Forest Region of Guinea (n = 1) and Sierra Leone (n = 1)). All subjects reached the sanctuary presenting varying states of mental and physical health. A four-point Likert scale was used to quantify the level of abnormal behaviors upon arrival; a minimum of five experienced staff members scored independently each individual they had personally witnessed arrive at the sanctuary (Table 2). The use of this simple four-point scale aimed to ensure that the staff could easily score each individual regardless of the individual’s date of arrival. Data were then averaged across all scorers to produce a final score for each individual ranging from zero to three [35] (Table 1). The low standard deviation reported in Table 1 confirmed there was a strong consensus among the scorers. The averaged abnormal behavior score upon arrival (ABS) was used as a covariate in our models. 

Once or twice daily, all CCC juveniles and infants go on bush-outings in the surrounding areas of the sanctuary. After being provisioned, the “Nursery” and the “TDT” chimpanzees are accompanied by one keeper and one to two volunteers during their bushwalks The CCC has a strict policy on human contact during bush-outings; contact typically only takes place when it is solicited and should not typically be initiated by a human caretaker unless necessary. 

**Table 1:** Summary of each subject’s sex, year of birth (YOB), current age (as of January 2012), age upon arrival to the Centre for Conservation of Chimpanzees (CCC), the number of years spent at sanctuary (as of January 2012), and average abnormal behavior likert-scale [35] score upon arrival (ABS).

Name	Sex	YOB	Group	Arrival Date (month/year)	Age of Individual (years)	Age at arrival (years)	Years at sanctuary	ABS ± 1SD
**HAKIM**	Male	2004	Nursery	04/06	8	2	6	0 ± 0.0
**AMA**	Female	2006	Nursery	05/08	6	2	4	1.6 ± 0.5
**KIRIKOU**	Male	2006	Nursery	05/08	6	2	4	2.2 ± 0.4
**LILI**	Female	2005	Nursery	03/07	7	2	5	0 ± 0.0
**FLO**	Female	2005	Nursery	08/07	7	2	5	0.75 ± 0.5
**DOUMA**	Male	2006	Nursery	06/07	6	1	5	0.25 ± 0.5
**PANZA**	Male	2005	Nursery	09/06	8	1	7	0.25 ± 0.5
**TANGO**	male	2009	TDT	06/10	4	1	3	0.2 ± 0.4
**TYA**	Female	2010	TDT	01/11	2	1	1	0.25 ± 0.5
**DEMOU**	Female	2007	TDT	03/11	5	4	1	0 ± 0.0

**Table 2:** Summary of scale used to quantify the degree of aberrant or stereotypic behaviors exhibited by each orphan upon arrival at the sanctuary. Experienced keepers, staff members and long-term volunteers who were present at the time were asked to score each chimpanzee independently. The final score for each individual was averaged based on a minimum of five scorers.

Likert Scale	Scale definition
**3**	Exhibits a range of abnormal behaviors, *i.e.*, intense rocking, hair plucking and/or self-mutilation, these behaviors are seen throughout the day under both stressful and relaxed situations/environments.
**2**	Exhibits abnormal behaviors occasionally under stressful and less stressful conditions, e.g., rocking, on a daily basis. Abnormal behaviors seen several times a day.
**1**	Exhibits abnormal behaviors on occasion and only under stressful situations. Abnormal behaviors observed no more than once a day.
**0**	No abnormal behaviors

### 2.2. Data Collection During Bush-Outings

Ten minute continuous individual focal follows were undertaken during bush-outings to record the duration of activities performed (total individual average time observed: “Nursery” = 799 ± 29.80 min; “TDT” = 1,053 ± 14.71 min) [36,37]. Each behavioral category was coded using a basic behavioral ethogram (Table 3). A change in behavioral state was recorded when the intervening interval surpassed 3 seconds. During focal follows, we also recorded the species and part of the food item consumed (Table 4). In addition, any physical contact with a human and its duration were recorded throughout the focal observations. Finally, we conjointly recorded continuously the duration of the arboreal or terrestrial location of the individual. All behavioral recordings were conducted by L. Ongman with a Palmtop recorder uploaded with Observer XT 10.1 (^®^Noldus Information Technology).

**Table 3:** Ethogram for data collection categories. All categories were recorded during focal follows, including behavior, location and human contact.

CATEGORIES	Definition
**BEHAVIORS**	
**Social Behavior**	An individual is engaged in social play or allo-grooming with humans or conspecifics. This category also includes rarely observed social sexual events.
**Feeding**	Individual eating a food item
**Searching for food **	An individual actively searching for food (may be collecting seed pods or fruit).
**Subsistence tool-use**	The use of an object, e.g., stick or stalk of vegetation, to probe or explore an opening, including termite mound holes, ground holes, or cracks. This behavioral category also included the use of a solid object to strike another to access potentially an embedded edible resource.
**General Solitary Behaviors**	Time spent self-grooming, playing alone or with an object, and solitary sexual events such as masturbation
**Observing**	Individual maintains gaze directed towards keeper or conspecific group member. Who is observed and their activity is also recorded. Gaze must be maintained for longer than 3 seconds and within 3 meters of the individual being observed.
**Nest Making**	Manipulation of branches and/or terrestrial herbaceous vegetation (THV) with the purpose to construct or modify a nesting structure either for resting or play.
**Travel**	Locomotion on the ground or in an arboreal setting (excludes individual displacement when actively searching for food—see above).
**Resting**	The individual is sleeping, standing or sitting and is not actively playing, grooming or partaking in any social behavior stated above, including not actively feeding, foraging, tool using or observing.
**LOCATION**	
**Arboreal**	Location of chimpanzee is in a tree or a vine, *i.e.*, off the ground.
**Terrestrial**	Location of chimpanzee is at ground level.
**HUMAN CONTACT**	
**Yes**	Individual is in direct physical contact with a human caretaker.
**No**	Individual is not in direct physical contact with a human caretaker.

**Table 4:** Plants and insects or insect products consumed during bush-outings by the “TDT” and “Nursery” groups between January 28–May 18 2012. Data includes species, family and part of plant eaten (THV: terrestrial herbaceous vegetation).

No.	Species	Family	Type of Plant	Part eaten
**1**	*Adansonia digitata*	Bombacaceae	Tree	fruit
**2**	*Afzelia Africana*	Caesalpinioideae	Tree	honey on leaves
**3**	*Allophyllus africanus*	Sapindaceae	Tree	fruit
**4**	*Andropogon gayanus*	Poaceae	Grass	leaf
**5**	*Bombax costatum*	Bombacaceae	Tree	flower, leaf
**6**	*Cassia sieberiana*	Caesalpinioideae	Tree	seed
**7**	*Carapa procera*	Meliaceae	Tree	fruit
**8**	*Cola cordifolia*	Sterculiaceae	Tree	flower, leaf stem, fruit, bark
**9**	*Cordia myxa*	Boraginaceae	Tree	fruit
**10**	*Costus afer*	Zingiberaceae	THV	stalk
**11**	*Daniella oliveri*	Caesalpinioideae	Tree	flower, seeds, new leaf
**12**	*Detarium microcarpum*	Caesalpinioideae	Tree	fruit, bark
**13**	*Dialium guineensis*	Caesalpinioideae	Tree	fruit
**14**	*Diospyros mespiliformis*	Ebenaceae	Tree	fruit, new leaf
**15**	*Dioscorea sp.*	Dioscoreaceae	Vine	leaf
**16**	*Ficus sp.*	Moraceae	Tree	fruit
**17**	*Ficus sur*	Moraceae	Tree	fruit
**18**	*Ficus thonninguii*	Moraceae	Tree	fruit
**19**	*Gardenia erubescens*	Rubiaceae	Tree	fruit
**20**	*Hanna undulate*	Simaroubaceae	Tree	fruit
**21**	*Khaya senegalensis*	Meliaceae	Tree	leaf, leaf stem
**22**	*Kigelia africana*	Bignoniaceae	Tree	fruit
**23**	*Landolphia heudelotti*	Apocynaceae	Vine	bark, fruit, leaf
**24**	*Lannea acida*	Anacardiaceae	Tree	seed
**25**	*Lannea microcarpa*	Anacardiaceae	Tree	leaf, bark
**26**	*Manguifera indica*	Anacardiaceae	Tree	leaf, fruit
**27**	*Marantochloa cuspidate*	Marantaceae	THV	stalk
**28**	*Opilia celtidifolia*	Opilianaceae	Vine	bark, leaf
**29**	*Oxytenanthera abyssinica*	Poaceae	Grass	stalk
**30**	*Parkia biglobosa*	Mimosaceae	Tree	flower, fruit
**31**	*Piliostigma thoningui*	Caesalpinioideae	Tree	seed
**32**	*Pterocarpus erinaceus*	Fabaceae	Tree	flower, new leaf
**33**	*Pterocarpus sp. santalinoides*	Fabaceae	Tree	flower
**34**	*Pterocarpus senegalensis*	Fabaceae	Tree	flower
**35**	*Raphia sp.*	Arecaceae	Tree	stalk
**36**	*Saba comorensis*	Apocynaceae	Vine	fruit
**37**	*Saba senegalensis*	Apocynaceae	Vine	fruit
**38**	*Siphonochilus sp.*	Zingiberaceae	THV	root, flower
**39**	*Smilax anceps*	Smilaceae	Vine	new leaf
**40**	*Tamarindus indica*	Caesalpinioideae	Tree	bark
**41**	*Uapaca somon*	Euphorbiaceae	Tree	fruit
**42**	*Ximenia Americana*	Olacaceae	Shrub	fruit
**43**	*Xylopia aethiopica*	Annonaceae	Tree	seed
**44**	*Oecophylla longinoda*	Formicidae	Insect	ant
**45**	*Procubitermes*	Termitidae	Insect	termite
**46**	*Trigona sp.*	Apidae	Honey	honey

### 2.3. Analysis

A Pearson’s correlation was performed on the percentage of time spent engaged in social behaviors (*i.e.*, social play and allo-grooming) and the percentage of time spent engaged in foraging (*i.e.*, eating, searching for food and subsistence tool use), in order to assess whether there was any behavioral trade-off between these two domains of behavior. Both percentages were calculated using the continuous focal data and the total time spent engaged in these activities was divided by the total focal time for each individual. 

Continuous focal follows served to calculate activity budgets across nine main behavioral state categories, *i.e.*, eating, searching for food, resting, traveling, observing conspecifics and humans, social behavior, solitary behavior as well as tool use and nest building (Table 3). The total duration of each activity was divided by the total focal time for each individual and computed as an overall mean percentage of activity. 

In order to analyze what variables predicted observation of conspecifics’ foraging and dietary knowledge, *i.e.*, the total number of food parts consumed by an individual, we used a general linear model (GLM) Univariate analysis in SPSS version 19. A Kolmogorov–Smirnov test was first run on all covariates and dependent variables to test the data for normality. Since all variables showed a normal distribution, all further statistical tests performed on the data were parametric. All tests were two-tailed and the significance level was p < 0.05. All means are reported with a ±1 standard deviation (SD) unless otherwise specified. 

The first GLM analysis examined the effects of individual age, sex and mean ABS on individual dietary knowledge. This model included additional covariates such as the amount of time individuals watched conspecifics foraging and percentage of time spent in contact with humans. Since the majority of food items consumed during the bush-outings occurred in an arboreal setting (see Result’s section: The overall percentage of food items which were located in an arboreal setting was 82%), the percentage of time spent arboreal was also examined in the model. Dietary knowledge was expected to be higher for older individuals. Individuals who spend more time in contact with humans were expected to present less dietary knowledge and to have exhibited higher levels of ABS. The second model explored whether the percentage of time spent observing conspecifics’ foraging was influenced by individual age, sex and the mean ABS as well as individual dietary knowledge and percentage of time spent foraging. More knowledgeable individuals, as reflected by their broader individual dietary repertoire, were expected to spend less time observing others, and individuals who spent more time foraging were expected to spend less time observing conspecifics’ foraging and *vice versa*. 

## 3. Results

Findings failed to show a significant correlation between percentage of time spent in social and foraging behaviors (Pearson correlation: n = 10, r = −0.537, p = 0.109). There was therefore no apparent trade-off at the individual level between time spent in one or the other of these domains of behavior.

### 3.1. Activity Budget, Location and Human Contact

**Figure 2:**
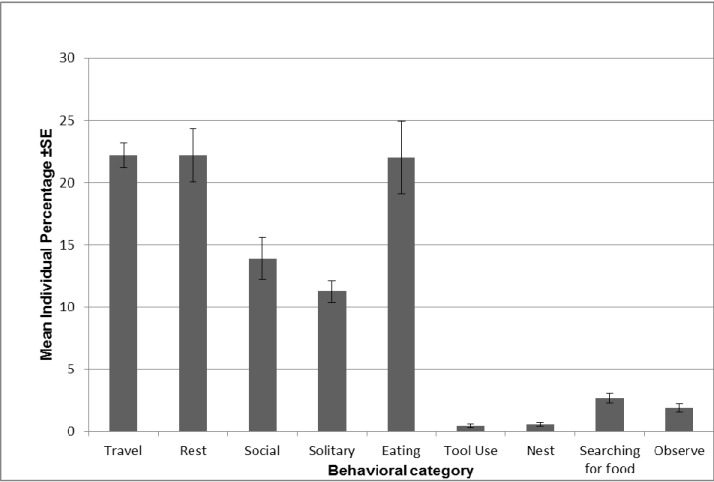
Mean individual percentage of time spent (±1 SE) across the nine main behavioral categories recorded during continuous focal follows.

When examining the mean individuals’ activity budget based on continuous focal follows, the chimpanzees on bush-outings spent on average nearly 70% of their time engaged in either resting (22.2 ± 6.7%), feeding (22.0 ± 9.3%) or traveling (22.6 ± 3.1%) (Figure 2). They spent a quarter of their time engaged in social (13.9 ± 5.3%) or solitary (11.2 ± 2.7%) behaviors, while tool use (0.5 ± 0.4%) and nesting behavior (0.6 ± 0.5%) occurred more rarely. The chimpanzees also spent a mean percentage of time of 1.5 ± 1.2% searching for food and 1.9 ± 1.1% observing others (Figure 2).

Based on the continuous focal data, individuals spent on average 55.5 ± 20.6% of their time terrestrial and 43.6 ± 21.2% of their time in an arboreal location. Also, individuals spent relatively less time in direct contact with human caretakers (19.0 ± 10.9%) than not (80.2 ± 10.9%).

### 3.2. Dietary Knowledge

**Figure 3:**
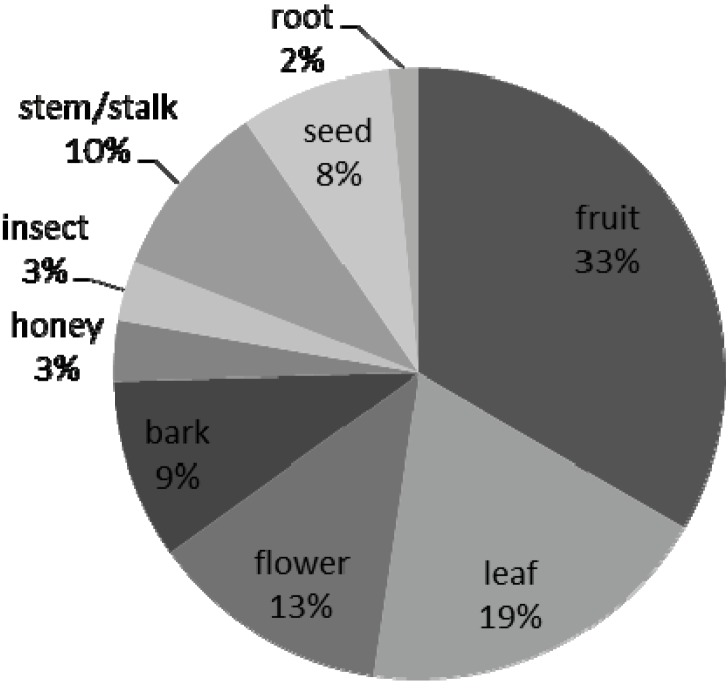
Percentage of food parts consumed (n = 63) during bush-outings during the four-month study period.

During this study, subjects consumed an individual mean of 27 ± 4.3 food items. The mean individual percentage of food items located and ingested in trees was 82 ± 6.5%. Data from focal follows revealed that the subjects of this study consumed altogether 46 different species including 63 different food items during the four-month study period (for full list see Table 4). The highest percentage of food parts consumed included fruit, leaves and flowers (33%, 19% and 13%, respectively); roots, insects and honey contributed the least to their overall dietary repertoire (Figure 3). The Univariate GLM model examined the influence of mean ABS, age, sex, as well as the percentage of time spent arboreal, in human contact, and observing conspecifics foraging upon individual variation in dietary knowledge. Only the percentage time spent arboreal emerged as significant (GLM: F_1,10_ = 10.27, p = 0.049).

Individual dietary knowledge and the percentage of time spent arboreal indeed showed a highly significant positive correlation (Pearson correlation: n = 10, r = 0.81, p = 0.005). Individuals who spent more time arboreal therefore showed a greater dietary knowledge than counterparts who spent more time on the ground. However, the percentage of time spent in human contact failed to correlate negatively with time spent arboreal (Pearson correlation: n = 10, r = −0.36, p = 0.31). Hence, contact with humans did not affect as predicted the chimpanzees’ ability to spend time arboreal.

### 3.3. Observing Conspecifics Foraging

During bush-outings individuals spent little of their total percentage time observing humans and conspecifics (Figure 2). The rehabilitants spent on average 54.4 ± 24.4% of their time observing conspecifics as a function of the total time spent observing others, *i.e.*, humans and conspecifics. Only 28.9 ± 24.9% of the total time observing others was spent observing conspecifics foraging. In addition, there were no recorded instances of individuals observing humans foraging, confirming that during this study social information on foraging could only be acquired via observation of conspecifics. Neither sex nor the covariates included in the GLM Univariate analysis, *i.e.*, age, individual mean ABS, individual dietary knowledge, individual percentage time spent foraging or in human contact, significantly explained the recorded variation in time spent observing conspecifics foraging. 

## 4. Discussion

### 4.1. Activity Budget

The percentage of time spent engaged in social play or allo-grooming did not correlate significantly with individual percentage time spent foraging. This finding suggests that CCC individuals showed no behavioral trade-off between these two core domains of activity. Our study rehabilitants spent on average slightly more time terrestrial than arboreal, although there was a great deal of variation across individuals. They also spent on average less time in direct contact with humans than not. The rehabilitant who spent the most time in human contact during this study happened to be the youngest and the newest arrival (2 years old with 46% of time spent in human contact).

Traveling, resting and foraging were the predominant activities recorded during bush-outings and contributed on average equally to the individuals’ activity budget. Although the chimpanzees were always fed before going out on bush-outings, they spent nearly a quarter of their time foraging (24.0 ± 10.9%). While provisioned, individuals were fed a balanced diet, which mirrored that of individuals who remained in cages or enclosures. Individuals who partook in bush-outings therefore continued to forage for wild foods either because they needed to supplement their provisioned diet and/or they preferred wild foods over provisioned foods. 

### 4.2. Dietary Knowledge

Our study period coincided with the end of the dry season, a period of fruit scarcity in the HNNP [38]. Nevertheless, during this study, fruit was the predominant food item consumed with as many as 21 different fruit species recorded as ingested, followed by leaves and flowers, with 12 and eight different plant species targeted respectively (Figure 3). High fruit consumption concurred with observations of wild chimpanzees recorded elsewhere [39]. The great majority of food items consumed were located up in the trees. We uncovered a significant positive correlation between the individual percentage time spent arboreal and dietary knowledge, which potentially highlights the significance of ecological opportunities in contributing to the expansion of individuals’ dietary knowledge. Unlike reported findings in Sumatran orangutans (*Pongo abelii*) [32], there was no effect of percentage time spent in human contact on individual dietary knowledge. Finally, contrary to expectation, neither age, sex nor mean ABS upon arrival predicted individual variation in dietary knowledge.

### 4.3. The Role of Observation

Although previous studies both in wild and captive settings revealed gender, age and rearing history effects on conspecific observation and individual social learning [19,28,40,41], none were detected in this study. In addition, while several studies have highlighted the importance of decreasing human contact to promote species-specific behaviors, especially for individuals rehabilitated for the purpose of release [12,32,42], contact with humans and human presence in the context of the CCC failed to predict potential observational learning of conspecifics engaged in foraging behavior. Albeit raised as an issue with rehabilitated orangutans [32], contact with humans during bush-outings did not affect the foraging development of CCC rehabilitants. However, during this study, rehabilitants spent a limited percentage of time observing others engaged in foraging activities. This low percentage may be attributed to the low frequency of the occurrence of complex food processing events during the study period, *i.e.*, the end of the dry season. Indeed, conspecific observation among young wild great apes is most often recorded when the ‘demonstrator’ is engaged in complex processing skills [31], such as nut cracking and ant dipping in chimpanzees [28,41]. Tool use at the CCC occurs predominantly during the rainy season due the availability of target foods, *i.e.*, driver ants *Dorylus* sp. or *Afzelia africana* pods which younger individuals crack with stones to access the seeds within during rainy season months [43]. Therefore, due to seasonality, there were fewer opportunities to gather data on subjects observing conspecifics performing complex foraging tasks such as tool use behaviors (Figure 2). Added to this, our definition of observation, *i.e.*, <3 m and observing an individual for more than 3 seconds, may be too narrow, precluding observation events occurring at greater distances or lasting for shorter durations. Hence, the role of observation in behavioral acquisition in our study may have been underestimated. Future studies should be performed over a longer sampling period to account for seasonality and across more subjects, and the definition of “observation” should potentially be revised to capture more effectively observation events of shorter duration and occurring at greater distances. Finally other behaviors, such as scrounging and co-feeding [30], should also be recorded to assess the contribution of other socially-biased learning mechanisms on dietary acquisition.

### 4.4. The “Super Chimpanzee” Theory

The dietary repertoire of our subjects (*i.e.*, 46 total number of species and 63 total food items consumed) was nearly equivalent to that reported in a three-year study of wild chimpanzees in Fongoli, Senegal, which registered the consumption of 47 different species and 60 different food items [18]. The latter site shares similar ecological conditions to the PNHN where the CCC is located [13,18,33]. Due to our comparatively short study period and smaller number of study subjects, the dietary knowledge of our study individuals and CCC rehabilitants as a whole could possibly far exceed that of Fongoli. If true, this difference could be attributed to a lower floristic diversity at Fongoli compared with the HNNP [13,18,33], but also to the unique conditions afforded by a sanctuary setting.

The relatively broad dietary knowledge recorded among the CCC subjects during our study may stem from two sanctuary-specific factors; *i.e.*, the young age of residents partaking in bush-outings and their different geographical origins. Most studies on wild chimpanzees suggest that social learning processes contribute the most to the acquisition of knowledge of edible foods and processing techniques, e.g., [25,28]. In this sense, the dietary knowledge of a group may be broadened (1) via individual acquisition of novel foods via trial and error (innovation) followed by social transmission (diffusion) or (2) the social transmission of new knowledge carried by migrant individuals (dissemination) [44]. Since young chimpanzees present a higher propensity for innovation and social learning than older subjects [28], the likelihood of innovation and behavioral diffusion may be higher among groups comprised of young rehabilitated orphans on bush-outings in an environment offering innovation opportunities than older individuals who are restricted to their enclosure. 

Cultural transmission has been documented in both wild and captive settings and this process can occur both within and between groups [28,31,45,46]. All subjects of our study and the majority of orphans at the CCC are wild-born and originate from different regions of Guinea or West Africa [47]. Each individual therefore carries with it its own environmental and cultural knowledge. Studies of wild chimpanzees have revealed that juveniles and infants, including individuals under 2 years old, are able to successfully perform tool use tasks such as using a probe to access an embedded food resource [41,46,48]. In addition, albeit not yet weaned, chimpanzees as young as 6 months old start to consume solid foods and are therefore beginning to build up familiarity with different edible food parts [49]. Therefore, in spite of an early separation from their mothers, young arrivals (average age of arrival for sample subjects was 1.8 years) likely possess dietary knowledge of their own and are familiar with different edible or non-edible food items. Since the environment around the CCC is highly heterogeneous [13] and many species recorded as consumed during this study are widely distributed across West Africa [50], some overlap in food species between the rehabilitants’ native environment and the CCC’s is highly probable. Rehabilitants could therefore be contributing their dietary knowledge additively to a pool of information of edible foods. Anecdotally, evidence of the ‘super chimpanzee’ theory was witnessed during our study when a recently arrived individual climbed an oil palm tree, *Elaeis guineensis,* and removed a frond to consume the petiole. Oil palm petiole feeding has been witnessed among wild chimpanzees elsewhere in Guinea and Africa [51]. No other individual in the group had ever been seen doing this behavior before. The following day, two other members were seen performing this behavior [52].

## 5. Conclusion

Until adolescence, the CCC offers the opportunity of bush-outings to its rehabilitants as daily enrichment. These bush-outings, during which human contact is principally initiated by the rehabilitants, provide highly stimulating settings offering learning opportunities and enabling the development of a range of behavioral skills. Daily bush-outings favor the chimpanzees’ immersion in natural surroundings where they may learn and perfect nest building skills, and broaden their dietary knowledge, as well as their social skills during interactions with conspecifics [13]. Neither sex nor age of our young subjects affected their rehabilitation performance in terms of their dietary knowledge. Our study also revealed that individuals with signs of psychological and emotional trauma upon arrival had an equivalent dietary knowledge compared to less traumatized orphans. Nonetheless, historical and latent stereotypic behaviors may serve to predict difficulties in the social domain [53]. Nevertheless, CCC staff have noted that, after several years of bush-outings, some chimpanzees ceased to demonstrate stereotypic behaviors, indicating that this form of rehabilitation can effectively help to curtail initial psychological and/or emotional trauma [54].

Data across sanctuaries, if gathered systematically using equivalent observational protocols and behavioral ethograms, could contribute to a better understanding of the key factors predicting rehabilitation success, individual progress and eventually release success. Collaborative research programs across sanctuaries and captive settings could therefore further help inform best rehabilitation practices [16,32,55]. For example, activity budget studies and evaluations of individual’s skillsets and behavior in both the physical and social domain could serve to compare rehabilitation approaches and environments both within and among sanctuaries. An activity budget may also monitor the evolution of individuals with respect to their history and personality, as well as group composition and dynamics. This study has begun to explore indicators and/or the learning and developmental processes underlying successful rehabilitation. These data can serve to inform future identification of suitable release candidates and also to help to improve the welfare of individual rehabilitants especially since the CCC keepers are now trained in behavioral data collection and are monitoring individuals. It is apparent that both social and ecological skills are required to maximize release success [12] and special attention should be given to both during rehabilitation since they are two distinct domains requiring differing skill sets. In addition, as proposed by others [12,42], there is a growing necessity for behavioral data on chimpanzees both at the individual and group level pre- and post-release to start identifying key behavioral indicators of release success. This study is a good start in the right direction. We hope to encourage more studies among rehabilitant chimpanzees aimed at better predicting and understanding rehabilitation and potentially release success among our closest living relatives.

## References

[1] Noon C. (1999). Chimpanzees and Retirement. J. Appl. Anim. Welf. Sci..

[2] Teleki G., Beck B., Stoinski T., Hutchins M., Maple T., Norton B., Rowan A., Stevens E., Arluke A. (2001). Sanctuaries for Ape Refugees. Great Apes and Humans, the Ethics of Coexistence.

[3] Farmer K. (2002). Pan-African Sanctuary Alliance: Status and Range of Activities for Great Ape Conservation. Am. J. Primatol..

[4] Campbell G., Kuehl H., N’Goran Kouamé P., Boesch C. (2008). Alarming Decline of West African Chimpanzees in Côte d’Ivoire. Curr. Biol..

[5] Schipper J., Chanson J.S., Chiozza F., Cox N.A., Hoffmann M., Katariya V., Lamoreux J., Rodrigues A.S., Stuart S.N., Temple H.J. (2008). The Status of the World’s Land and Marine Mammals: Diversity, Threat, and Knowledge. Science.

[6] Beck B., Lonsdorf E., Ross S., Matsuzawa T. (2010). Chimpanzee Orphans: Sanctuaries, Reintroduction and Cognition. The Mind of the Chimpanzee: Ecological and Experimental Perspectives.

[7] Morimura N., Idani G., Matsuzawa T. (2011). The First Chimpanzee Sanctuary in Japan: An Attempt to Care for the “Surplus” of Biomedical Research. Am. J. Primatol..

[8] Faust L., Cress D., Farmer K., Ross S., Beck B. (2011). Predicting Capacity Demand on Sanctuaries for African Chimpanzees (*Pan troglodytes*). Int. J. Primatol..

[9] Beck B., Walkup K., Rodrigues M., Unwin S., et Tara Stoinski D.T. (2007). Best Practice Guidelines for the Reintroduction of Great Apes.

[10] Hannah A., McGrew W. (2005). Rehabilitation of Captive Chimpanzees. Primate Responses to Environmental Change.

[11] Yeager C. (1997). Orangutan Rehabilitation in Tanjung Puting National Park, Indonesia. Conserv. Biol..

[12] Farmer K., Honig N., Goossens B., Jamart A., Soorae P. (2010). Habitat Ecologique et Liberte des Primates: Re-Introduction of Central Chimpanzees to the Conkouati-Douli National Park, Republic of Congo. IUCN/SSC: Global Re-Introduction Perspectives: Additional Case-Studies from Around the Globe.

[13] Humle T., Colin C., Laurans M., Raballand E. (2010). Group Release of Sanctuary Chimpanzees (*Pan troglodytes*) in the Haut Niger National Park, Guinea, West Africa: Ranging Patterns and Lessons So Far. Int. J. Primatol..

[14] Reimers M., Schwarzenberger F., Preuschoft S. (2007). Rehabilitation of Research Chimpanzees: Stress and Coping after Long-Term Isolation. Hormones Behav..

[15] Fritz J., Maki S., Nash L., Martin T., Matevia M. (1992). The Relationship Between Forage Material and Levels of Coprophagy in Captive Chimpanzees (*Pan troglodytes*). Zoo Biol..

[16] Wobber V., Hare B. (2011). Psychological Health of Orphan Bonobos and Chimpanzees in African Sanctuaries. PLoS ONE.

[17] Doran D. (1997). Influence of Seasonality on Activity Patterns, Feeding Behavior, Ranging, and Grouping Patterns in Taï Chimpanzees. Int. J. Primatol..

[18] Preutz J., Hohmann G., Robbins M., Boesch C. (2006). Feeding Ecology of Savanna Chimpanzees (*Pan troglodytes* verus) in Fongoli, Senegal. Feeding Ecology in Apes and Other Primates: Ecological, Physical, and Behavioral Aspects.

[19] Goodall J. (1986). The Chimpanzees of Gombe: Patterns of Behavior.

[20] Boesch C., Boesch H. (1989). Hunting Behavior of Wild Chimpanzees in the Taï National Park. Am. J. Phys. Anthropol..

[21] Yamakoshi G. (1998). Dietary Responses to Fruit Scarcity of Wild Chimpanzees at Bossou, Guinea: Possible Implications for Ecological Importance of Tool Use. Am. J. Phys. Anthropol..

[22] Marshall A., Wrangham R. (2007). Evolutionary Consequences of Fallback Foods. Int. J. Primatol..

[23] van-Lawick-Goodall J., Menzel E.W. (1973). Cultural Elements in a Chimpanzee Community. Precultural Primate Behavior.

[24] Pusey A. (1983). Mother-Offspring Relationships in Chimpanzees after Weaning. Anim. Behav..

[25] Hiraiwa-Hasegawa M., Heltne P., Marquardt L. (1989). Sex Differences in the Behavioral Development of Chimpanzees at Mahale. Understanding Chimpanzees.

[26] Van Schaik C. (2003). Orangutan Cultures and the Evolution of Material Culture. Science.

[27] Galef B., Giraldeau L. (2001). Social Influences on Foraging in Vertebrates: Causal Mechanisms and Adaptive Functions. Anim. Behav..

[28] Biro D., Inoue-Nakamura N., Tonooka R., Yamakoshi G., Sousa C., Matsuzawa T. (2003). Cultural Innovation and Transmission of Tool Use in Wild Chimpanzees: Evidence from Field Experiments. Anim. Cognit..

[29] Lonsdorf E. (2005). Sex Differences in the Development of Termite-Fishing Skills in the Wild Chimpanzees, (*Pan troglodytes schweinfurthii*), of Gombe National Park, Tanzania. Anim. Behav..

[30] Rapaport L., Brown G. (2008). Social Influences on Foraging Behavior in Young Nonhuman Primates: Learning What, Where, and How to Eat. Evol. Anthropol. Issues News Rev..

[31] Jaeggi A., Dunkel L., Van Noordwijk M., Wich S., Sura A., Van Schaik C. (2010). Social Learning of Diet and Foraging Skills by Wild Immature Bornean Orangutans: Implications for Culture. Am. J. Primatol..

[32] Riedler B., Millesi E., Pratje P. (2010). Adaptation to Forest Life During the Reintroduction Process of Immature *Pongo abelii*. Int. J. Primatol..

[33] Brugiere D., Dia M., Diakite S., Gbansara M., Mamy M., Saliou B., Magassouba B. (2005). Large- and Medium-Sized Ungulates in the Haut Niger National Park, Republic of Guinea: Population Changes 1997–2002. Oryx.

[34] Fleury-Brugiere M., Brugiere D. (2010). High Population Density of *Pan troglodytes verus* in the Haut Niger National Park, Republic of Guinea: Implications for Local and Regional Conservation. Int. J. Primatol..

[35] Likert R. (1932). A Technique for the Measurement of Attitudes. Arch. Psychol..

[36] Altmann J. (1974). Observational Study of Behavior: Sampling Methods. Behaviour.

[37] Martin P., Bateson P. (2009). Measuring Behaviour: An Introductory Guide.

[38] Humle T. (2008).

[39] Humle T., Matsuzawa T., Humle T., Sugiyama Y., Matsuzawa T., Yamagiwa J. (2011). Location and Ecology. The Chimpanzees of Bossou and Nimba.

[40] Boesch C., Boesch-Achermann H. (2000). The Chimpanzees of the Taï Forest: Behavioural Ecology and Evolution.

[41] Humle T., Snowdon C., Matsuzawa T. (2009). Social Influences on Ant-Dipping Acquisition in the Wild Chimpanzees (*Pan troglodytes verus*) of Bossou, Guinea, West Africa. Anim. Cognit..

[42] Baker K. (2002). Guidelines for Nonhuman Primate Re-Introductions. Re-Introduction News.

[43] Humle T., Ongman L. (2012). Personal observation.

[44] Humle T., Hicks D., Beaudry M. (2010). Primate Material Culture. The Oxford Handbook of Material Culture Studies.

[45] Whiten A., Spiteri A., Horner V., Bonnie K., Lambeth S., Schapiro S., de Waal F. (2007). Transmission of Multiple Traditions within and between Chimpanzee Groups. Curr. Biol..

[46] Humle T., Snowdon C. (2008). Socially Biased Learning in the Acquisition of a Complex Foraging Task in Juvenile Cottontop Tamarins, (*Saguinus oedipus*). Anim. Behav..

[47] Chimpanzee Conservation Center (2012).

[48] Lonsdorf E. (2006). What is the Role of Mothers in the Acquisition of Termite-Fishing Behaviors in Wild Chimpanzees (*Pan troglodytes schweinfurthii*)?. Anim. Cognit..

[49] Nishida T., Turner L. (1996). Food Transfer Between Mother and Infant Chimpanzees of the Mahale Mountains National Park, Tanzania. Int. J. Primatol..

[50] Hawthorne W., Jongkind C. (2006). Woody Plants of Western African Forests.

[51] Humle T., Matsuzawa T. (2004). Oil Palm Use by Adjacent Communities of Chimpanzees at Bossou and Nimba Mountains, West Africa. Int. J. Primatol..

[52] Ongman L. (2012). Personal observation.

[53] Ongman L., Colin C., Raballand E., Humle T. (2013). Individual History and the Social Dimensions of Rehabilitation in Chimpanzees.

[54] Raballand E., Colin C. (2012). Personal observation.

[55] Farmer K., Buchanan-Smith H.M., Jamart A. (2006). Behavioral Adaptation of *Pan troglodytes troglodytes*. Int. J. Primatol..

